# Maternal pre-pregnancy body mass index and newborn telomere length

**DOI:** 10.1186/s12916-016-0689-0

**Published:** 2016-10-18

**Authors:** Dries S. Martens, Michelle Plusquin, Wilfried Gyselaers, Immaculata De Vivo, Tim S. Nawrot

**Affiliations:** 1 Centre for Environmental Sciences, Hasselt University, Hasselt, 3500 Belgium; 2 MRC/PHE Centre for Environment and Health, School of Public Health, Imperial College London, London, W2 1PG UK; 3 Department of Obstetrics, East-Limburg Hospital, Genk, 3600 Belgium; 4 Channing Division of Network Medicine, Department of Medicine, Brigham and Women’s Hospital and Harvard Medical School, Boston, MA 02215 USA; 5 Program in Genetic Epidemiology and Statistical Genetics, Harvard School of Public Health, Boston, MA 02115 USA; 6 Department of Public Health & Primary Care, Leuven University, Leuven, 3000 Belgium

**Keywords:** Telomeres, Newborns, Pre-pregnancy body-mass index, *in utero* life

## Abstract

**Background:**

Newborn telomere length sets telomere length for later life. At birth, telomere length is highly variable among newborns and the environmental factors during *in utero* life for this observation remain largely unidentified. Obesity during pregnancy might reflect an adverse nutritional status affecting pregnancy and offspring outcomes, but the association of maternal pre-pregnancy body mass index (BMI) with newborn telomere length, as a mechanism of maternal obesity, on the next generation has not been addressed.

**Methods:**

Average relative telomere lengths were measured in cord blood (*n* = 743) and placental tissue (*n* = 702) samples using a quantitative real-time PCR method from newborns from the ENVIR*ON*AGE birth cohort in Belgium. By using univariate and multivariable adjusted linear regression models we addressed the associations between pre-pregnancy BMI and cord blood and placental telomere lengths.

**Results:**

Maternal age was 29.1 years (range, 17–44) and mean (SD) pre-pregnancy BMI was 24.1 (4.1) kg/m^2^. Decline in newborn telomere length occurred in parallel with higher maternal pre-pregnancy BMI. Independent of maternal and paternal age at birth, maternal education, gestational age, newborn gender, ethnicity, birthweight, maternal smoking status, parity, cesarean section, and pregnancy complications, each kg/m^2^ increase in pre-pregnancy BMI was associated with a −0.50 % (95 % CI, −0.83 to −0.17 %; *P* = 0.003) shorter cord blood telomere length and a −0.66 % (95 % CI, −1.06 to −0.25 %; *P* = 0.002) shorter placental telomere length.

**Conclusions:**

Maternal pre-pregnancy BMI is associated with shorter newborn telomere lengths as reflected by cord blood and placental telomeres. These findings support the benefits of a pre-pregnancy healthy weight for promoting molecular longevity from early life onwards.

**Electronic supplementary material:**

The online version of this article (doi:10.1186/s12916-016-0689-0) contains supplementary material, which is available to authorized users.

## Background

The prevalence of overweight and obesity is rising worldwide, including in women of reproductive age. Maternal overweight and obesity are well-known risk factors for adverse pregnancy outcomes and maternal body mass index (BMI) is also associated with maternal blood glucose levels during pregnancy [1]. Higher risks for pregnancy complications, such as preeclampsia [2], gestational diabetes [3], hypertensive disorders [4], and cesarean delivery [5], have been associated with maternal obesity. Telomeres are nucleoprotein structures containing TTAGGG repeats at the end of the chromosomes. They are important in maintaining genomic stability and protect chromosomes from end-to-end fusion and degradation [6]. Telomere length is considered a biomarker of biological ageing and has been associated with age-related diseases such as cardiovascular disease [7, 8], type 2 diabetes [9], atherosclerosis [10, 11], and increased mortality [12–14]. Telomere length variability and attrition rate has been explained by heritability and by different environmental determinants [15–19]. Even at birth newborn telomere lengths are highly variable [20, 21] and the rate of telomere shortening is higher during the first 4 years of life compared to later life [22].

Environmental factors during *in utero* life that potentially explain the observed telomere length variability in newborns remain largely unidentified since large population-based studies of telomere lengths in relation with environmental factors and age-related diseases have traditionally recruited middle-aged subjects [23, 24]. Nevertheless, interest of *in utero* factors (physiological, social, environmental, demographical, and clinical exposures during pregnancy) in relation to newborn telomere biology is rising [24]. Indeed, recent studies have shown that newborn telomere length is influenced by intrauterine effects such as exposure to maternal stress [25, 26], maternal smoking [27], maternal education [28], maternal folate concentrations [29], pre-eclampsia, and intrauterine growth restriction [30, 31], as well as by diabetes during pregnancy [32]. These findings reflect the importance of *in utero* and early life exposures in the determination of initial telomere lengths at birth and show that these exposures may have an impact on ageing, disease susceptibility, and molecular longevity later in life. Telomere length in adulthood is associated with BMI [33], but the influence of pre-pregnancy weight through the intrauterine environment on telomere length has not been addressed. Maternal undernutrition during *in utero* development of the fetus has been associated with coronary heart disorders [34], increased BMI [35, 36], and hypertension [35, 37]. During pregnancy, maternal obesity might influence the *in utero* environment, which can lead to altered fetal development, physiology, and metabolism, potentially underlying the origin of later life diseases and possibly having an impact on later life health [38]. Maternal obesity is associated with abnormal fetal growth [39], increased risks of birth defects [40, 41], fetal death, still birth, and infant death [42]. Later life health states, such as childhood obesity [43], childhood asthma [44], and cardiovascular diseases [45, 46], are associated with maternal obesity. These observations are consistent with the developmental origin of disease hypothesis [47]. We hypothesized that maternal overweight and obesity during pregnancy might be important in setting telomere length at birth and may therefore contribute to the developmental programming of the child. The aim of the present study was to evaluate the possible effects of maternal obesity in a representative cohort of newborns on telomere length measured in fetal cord blood DNA and placental DNA.

## Methods

### Study population and data collection

This study included 768 mother-newborn pairs (singletons) selected from the ongoing ENVIR*ON*AGE (ENVIRonmental influence *ON* AGEing in early life) birth cohort in the province of Limburg in Belgium. The study protocol was approved by the Ethical Committee of Hasselt University and East-Limburg Hospital in Genk (Belgium) and has been carried out according to the declaration of Helsinki. Written informed consent was obtained from all participating mothers. In total, 768 mothers with a singleton pregnancy and all with a pre-pregnancy BMI below 40 kg/m^2^ were recruited from February 1, 2010, to February 1, 2015, between Friday 1200 hours and Monday 0700 hours. The mother’s ability to fill out questionnaires in Dutch was a criterion for selection. The overall participation rate was 61 %. Because DNA was missing or of bad quality for cord blood DNA (*n* = 14) or for placental DNA (*n* = 57) or because telomere length measurements were too variable between triplicate measurements for cord blood telomere (*n* = 10) or placental telomere (*n* = 8), and because of missing data on maternal weight gain during pregnancy (*n* = 1), the final sample consisted of 743 mother-newborn pairs to study associations with cord blood telomere lengths and 702 mother-newborn pairs to study associations with placental telomeres (Additional file 1: Figure S1).

Data on maternal pre-pregnancy weight, weight before delivery, and height were collected from the medical records at the hospital. Maternal height and weight were measured without shoes, wearing light clothes to the nearest centimeter and weight to the nearest 0.1 kg at the first antenatal visit of each pregnancy (weeks 7–9 of gestation). BMI was defined as weight in kilograms divided by the square of height in meters. Gestational age was estimated based on ultrasound data. Pre-pregnancy BMI was categorized into three categories; normal was defined as BMI below 25 kg/m^2^, overweight when BMI was greater than or equal to 25 kg/m^2^ and below 30 kg/m^2^, and obese was defined when BMI was greater than or equal to 30 kg/m^2^. Furthermore, the women were weighed on admission to the delivery ward. Maternal pregnancy weight gain (weight before delivery minus pre-pregnancy weight) was categorized according to the Institute of Medicine guidelines – we defined insufficient and excessive gestational weight gain in relation to maternal pre-pregnancy BMI (for underweight: total weight gain < 13 and > 18 kg; normal weight: total weight gain < 11.5 and > 16.0 kg; for overweight: total weight gain < 7.0 and > 11.5 kg; for obesity: total weight gain < 5.0 and > 9.0 kg, respectively) [48].

Study questionnaires were completed in the post-delivery ward to provide detailed information on maternal age, paternal age, maternal education (as a measure for socioeconomic status), smoking status, parity, ethnicity, and pregnancy complications. Maternal smoking status was assessed as former smokers who had quit smoking before pregnancy and smokers who continued smoking during pregnancy. Maternal education was coded low when mothers only went to primary school and did not obtain a diploma, middle as they obtained a high school diploma and high when they obtained a college or university degree.

Newborn’s ethnicity was classified as European-Caucasian when two or more grandparents were European or non-European when at least three grandparents were of non-European origin. Information on pregnancy complications were collected from the medical records for each mother. Pregnancy complications was coded as absent if mothers did not experience any pregnancy complications or as present if mothers experienced one or more pregnancy complications. Included pregnancy complications were gestational diabetes, hypertension, infection diseases, pre-eclampsia, vaginal bleeding, and hyper- or hypothyroidism. Perinatal parameters were obtained after birth such as birth date, newborn gender, birth weight, and Apgar score. The ENVIR*ON*AGE birth cohort is generalizable to the gestational segment of the population at large as it did not differ from all births in Flanders as to maternal age, education, parity, newborn gender, ethnicity, and birth weight (Additional file 1: Table S1) [49].

### Cord blood and placental tissue collection

Umbilical cord blood was drawn immediately after delivery in BD Vacutainer® plastic whole blood tubes with spray-coated K2EDTA (BD, Franklin Lakes, NJ, USA). Samples were centrifuged at 3200 rpm for 15 min and blood tubes were stored at −80 °C. Buffy coat was removed upon DNA extraction. Placentas were collected and stored at −20 °C within 10 minutes after delivery. Placental biopsies were taken at the fetal site and stored at −80 °C upon DNA extraction as described previously [50]. For each placenta, four different biopsies were taken at four standardized sites across the middle region of the placenta, at 4 cm approximately from the umbilical cord. First, we determined within-placental average relative telomere length variation in 14 different placentas based on the four different biopsies. This average within placental variation was 11.7 %. Because of the low variation in telomere length within the placenta for different biopsies we used only one biopsy (1–2 cm^3^) taken to the right of the main artery for placental telomere length assessment.

### Average relative telomere length measurement

DNA was extracted from cord blood buffy coat and placental tissue using the QIAamp DNA Mini Kit (Qiagen, Inc., Venlo, The Netherlands). DNA quantity and purity was assessed by a Nanodrop 1000 spectrophotometer (Isogen, Life Science, Belgium). DNA integrity was assessed by agarose gel-electrophoresis. Average relative telomere length was measured by a modified quantitative real-time PCR (qPCR) protocol as described previously [51]. To ensure a uniform DNA input of 5 ng for each qPCR reaction, samples were diluted and checked using the Quant-iT™ PicoGreen® dsDNA Assay Kit (Life Technologies, Europe). Telomere and single copy-gene reaction mixture and PCR cycles used are given in Additional file 1: Text S1. All measurements were performed in triplicate on a 7900HT Fast Real-Time PCR System (Applied Biosystems) in a 384-well format. On each run, a 6-point serial dilution of pooled buffy coat or pooled placental DNA was run to assess PCR efficiency as well as eight inter-run calibrators to account for inter-run variability. Relative average telomere lengths were calculated using qBase software (Biogazelle, Zwijnaarde, Belgium) and were expressed as the ratio of telomere copy number to single-copy gene number (T/S) relative to the average T/S ratio of the entire sample set. Before our study, we performed an interlaboratory comparison of our telomere assay with a US reference lab to standardize the protocol. We achieved coefficients of variation (CV) within triplicates of the telomere runs, single-copy gene runs, and T/S ratios of 0.68 %, 0.41 %, and 6.4 %, respectively, for cord blood telomeres. For placental telomeres, we achieved CVs of 0.70 %, 0.45 %, and 6.9 %, for telomere runs, single-copy gene runs, and T/S ratios, respectively.

### Statistical analysis

All statistical analyses were performed using the SAS 9.3 statistical software (SAS Institute Inc., Cary, NC, USA). Continuous variables were tested for normality. Average relative cord blood and placental telomere lengths showed skewed distribution and were log10 transformed to improve normal distribution. To study potential confounding structure in our dataset, we assessed the distributions of continuous variables (ANOVA) and proportions of categorical variables (χ^2^ test) across different classes of maternal pre-pregnancy BMI (normal, overweight, and obese). Pearson correlation (unadjusted analysis) and multiple linear regressions were applied to address the association between maternal pre-pregnancy BMI and cord blood or placental telomere lengths. In a first model, we adjusted for maternal and paternal age, maternal education, newborn gender, gestational age and birth weight. In a second model, we additionally adjusted for parity, maternal smoking status, newborn ethnicity, cesarean section, and pregnancy complications. Finally, we introduced maternal net weight gain during pregnancy to our models as a continuous variable as well as a categorical variable coded as sufficient and insufficient weight gain using the definition of Institute of Medicine guidelines (see Study population and data collection). In order to test whether the association of gestational weight gain on newborn telomere length is influenced by maternal pre-pregnancy BMI, we tested the interaction between weight gain and pre-pregnancy BMI. We ran different sensitivity analyses in which we separately excluded mothers from non-European origin, those with pre-pregnancy BMI less than 18.5, those who underwent cesarean section, or those who experienced complications during pregnancy.

## Results

### Study population characteristics

Demographic characteristics and perinatal factors of 743 mother-newborn pairs are reported in Table 1. Briefly, mean maternal age was 29.1 years (range, 17–44) and mean (SD) pre-pregnancy BMI was 24.1 (4.1) kg/m^2^. Of the 743 mothers, 175 (23 %) were overweight and 80 (11 %) were obese. Most women (63 %, *n* = 465) never smoked cigarettes and 178 women (24 %) stopped smoking before pregnancy, whereas 100 mothers (13 %) reported to have smoked during pregnancy (on average 8.6 cigarettes per day). The newborns, among them 376 girls (51 %), had a mean gestational age of 39.2 weeks (range, 30–42) and comprised 417 (56 %) primiparous and 249 (34 %) secundiparous newborns. About 89 % (*n* = 658) of the newborns were Europeans of Caucasian ethnicity. The mean (SD) birth weight of the newborns was 3400 (475) g. Five minutes after delivery, more than 90 % of the newborns had an Apgar score of 9 or 10. Higher maternal pre-pregnancy BMI was associated with more cesarean sections (*P* for trend = 0.005), pregnancy complications (*P* for trend = 0.006), and higher newborn birth weight (*P* for trend = 0.0005). Obese mothers had gained less weight during pregnancy compared to normal weight and overweight mothers (*P* for trend < 0.0001). The percentage of newborns from non-European origin was somewhat higher in the highest versus lowest BMI class (17 % vs. 10 %).

**Table 1:** Mother-newborn characteristics according to maternal pre-pregnancy BMI

Characteristic	Normal weight *n* = 488	Overweight *n* = 175	Obese *n* = 80	*P* for trend
Newborn				
Gender				
Girls, n	251 (51.4 %)	84 (48.0 %)	41 (51.3 %)	0.73
European-Caucasian, n	441 (90.4 %)	151 (86.3 %)	66 (82.5 %)	0.07
Gestational age, weeks	39.1 (1.5)	39.3 (1.4)	39.3 (1.7)	0.27
Birth weight, g	3351 (468)	3490 (453)	3502 (524)	0.0005
Maternal				
Age, years	29.2 (4.5)	29.3 (4.6)	28.4 (4.5)	0.34
Education				
Low, n	54 (11.1 %)	18 (10.3 %)	11 (13.8 %)	0.25
Middle, n	170 (34.8 %)	70 (40.0 %)	36 (45.0 %)	
High, n	264 (54.1 %)	87 (49.7 %)	33 (41.3 %)	
Smoking status				
Never smoker, n	305 (62.5 %)	109 (62.3 %)	51 (63.8 %)	0.80
Stopped smoker, n	118 (24.2 %)	39 (22.3 %)	21 (26.3 %)	
Continued smoker, n	65 (13.3 %)	27 (15.4 %)	8 (10.0 %)	
Cesarean section, n	13 (2.7 %)	13 (7.4 %)	7 (8.8 %)	0.005
Pregnancy complication, n	38 (7.8 %)	23 (13.1 %)	17 (21.3 %)	0.006
Parity				
1, n	285 (58.4 %)	86 (49.1 %)	46 (57.5 %)	0.12
2, n	158 (32.4 %)	63 (36.0 %)	28 (35.0 %)	
≥3, n	45 (9.2 %)	26 (14.9 %)	6 (7.5 %)	
Pregnancy weight gain, kg	14.9 (5.2)	14.1 (6.5)	11.4 (6.1)	<0.0001
Paternal				
Age, years	31.7 (4.9)	31.7 (4.7)	31.1 (5.7)	0.59

Classification of pre-pregnancy BMI classes: Normal when BMI < 25 kg/m^2^, overweight when 25 ≤ BMI < 30 kg/m^2^, and obese when BMI > 30 kg/m^2^

Values are presented as means (SD) or n (%)

### Predictors of newborn telomere length

The relative telomere length ranged from 0.51 to 1.75 in cord blood and 0.52 to 1.89 in placental tissue. The cord placental telomere length correlation was 0.44 (*P* < 0.0001) (Additional file 1: Figure S2). Compared with newborn boys, newborn girls had 6.83 % (95 % CI, 4.27–9.30 %; *P* < 0.0001) longer telomere length in cord blood and 5.24 % (95 % CI, 2.05–8.32 %; *P* = 0.002) longer in placental tissue. Low maternal education was associated with shorter cord blood telomeres (−6.04 %; 95 % CI, −10.18 to −1.71 %; *P* = 0.007) when comparing to the highest maternal education level. Cord blood and placental telomere length increased with 0.27 % (95 % CI, −0.01 to 0.54 %; *P* = 0.06) and 0.25 % (95 % CI, −0.08 to 0.59 %; *P* = 0.14), respectively, for each year increase in paternal age. No significant associations were found with cord blood or placental telomeres and maternal age, maternal weight gain during pregnancy, gestational age, maternal smoking status, newborn ethnicity, pregnancy complications, or delivery modus.

### Association between pre-pregnancy BMI and newborn telomere length

**Fig. 1:**
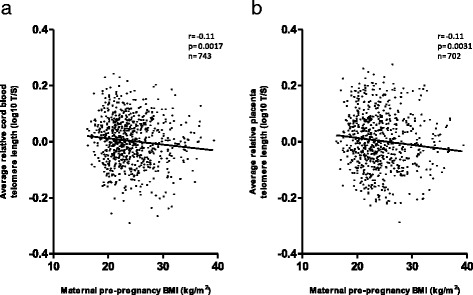
Pearson correlation between maternal pre-pregnancy BMI and newborn telomere length. Relative average telomere lengths were expressed as the ratio of telomere copy number to single-copy gene number (T/S ratio). **a** Cord blood telomeres. **b** Placental telomeres

Compared to mothers with a normal weight, cord blood and placental telomere length were lower in overweight and obese women (Table 2). In continuous analysis, both before (Fig. 1) and after mutual adjustment for different sets of covariates and potential confounders, both cord blood and placental telomere length (Table 3) were consistently lower with higher pre-pregnancy BMI. For each unit (1 kg/m^2^) increment in maternal pre-pregnancy BMI, cord blood telomere length was 0.50 % shorter (95 % CI, −0.83 to −0.17 %; *P* = 0.003) and placental telomere length was 0.66 % shorter (95 % CI, −1.06 to −0.25 %; *P* = 0.002). Mothers gained weight during pregnancy on the average (14.3 ± 5.6 kg) but further cumulative adjustment for maternal weight gain did not influence our results (Table 4). We did not observe a weight gain during pregnancy by pre-pregnancy BMI interaction with newborn cord (*P* = 0.85) or placental (*P* = 0.22) telomere length. Categorical analysis showed no association of insufficient and sufficient weight gain during pregnancy on newborn telomere length (Table 4). Sensitivity analyses with exclusion of newborns from non-European origin, mothers with pre-pregnancy BMI less than 18.5, mothers that underwent cesarean sections, or experienced pregnancy complications did not materially alter our results (Table 5).

**Table 2:** Categorized pre-pregnancy maternal BMI and newborn telomere length

	Cord blood (*n* = 743)		Placenta (*n* = 702)	
	Percentage change (95 % CI)	*P* value	Percentage change (95 % CI)	*P* value
Unadjusted				
<25 kg/m^2 a^	Ref		Ref	
Overweight^b^	−3.0 (−6.1 to 0.3)	0.07	−4.2 (−7.9 to −0.3)	0.03
Obese^c^	−5.7 (−9.8 to −1.3)	0.01	−3.7 (−8.8 to 1.6)	0.17
Overweight + Obese	−3.8 (−6.5 to −1.0)	0.008	−4.1 (−7.3 to −0.7)	0.02
Model A				
<25 kg/m^2^	Ref		Ref	
Overweight^b^	−2.5 (−5.6 to 0.8)	0.13	−4.2 (−7.8 to −0.3)	0.04
Obese^c^	−5.2 (−9.3 to −0.9)	0.02	−3.6 (−8.7 to 1.8)	0.19
Overweight + Obese	−3.3 (−6.0 to −0.5)	0.02	−4.0 (−7.3 to −0.6)	0.02
Model B				
<25 kg/m^2^	Ref		Ref	
Overweight^b^	−2.5 (−5.6 to 0.8)	0.14	−4.6 (−8.4 to −0.7)	0.02
Obese^c^	−5.5 (−9.7 to −1.1)	0.01	−4.2 (−9.3 to 1.3)	0.13
Overweight + Obese	−3.4 (−6.2 to −0.5)	0.01	−4.5 (−7.8 to −1.0)	0.01

Estimates are presented as a percentage change in average relative telomere length

Model A, adjustment for paternal and maternal age at birth, maternal education, newborn gender, gestational age, and birth weight. Model B, additionally adjusted for maternal smoking status, parity, newborn ethnicity, pregnancy complications, and cesarean section

^a^ *n* = 488 for cord blood and *n* = 455 for placenta

^b^ *n* = 175 for cord blood and *n* = 170 for placenta

^c^ *n* = 80 for cord blood and *n* = 77 for placenta

**Table 3:** Pre-pregnancy maternal BMI and newborn telomere length

	Cord blood telomere length (*n* = 743)		Placental telomere length (*n* = 702)	
	Percentage change (95 % CI)	*P* value	Percentage change (95 % CI)	*P* value
Unadjusted model	−0.52 (−0.85 to −0.20)	0.002	−0.60 (−0.99 to −0.20)	0.003
Model A	−0.48 (−0.80 to −0.15)	0.004	−0.60 (−1.00 to −0.20)	0.003
Model B	−0.50 (−0.83 to −0.17)	0.003	−0.66 (−1.06 to −0.25)	0.002

Estimates are presented as a percentage change in average relative telomere length for each kg/m^2^ BMI increase

Model A, adjustment for paternal and maternal age at birth, maternal education, newborn gender, gestational age, and birth weight

Model B, additionally adjusted for maternal smoking status, parity, newborn ethnicity, pregnancy complications, and cesarean section

**Table 4:** Pre-pregnancy maternal BMI, weight gain during pregnancy, and newborn telomere length

	Cord blood (*n* = 743)		Placenta (*n* = 702)	
	Percentage change (95 % CI)	*P* value	Percentage change (95 % CI)	*P* value
Weight gain continuous				
BMI, +1 kg/m^2^	−0.51 (−0.85 to −0.17)	0.003	−0.64 (−1.06 to −0.23)	0.002
Weight gain, +5.6 kg	−0.25 (−1.69 to 1.20)	0.73	0.20 (−1.59 to 2.02)	0.83
Weight gain categorical				
BMI, +1 kg/m^2^	−0.51 (−0.84 to −0.17)	0.003	−0.68 (−1.09 to −0.27)	0.001
Sufficient	Ref		Ref	
Insufficient	2.07 (−1.77 to 6.07)	0.29	3.01 (−1.77 to 8.03)	0.22
Excessive	1.55 (−1.58 to 4.78)	0.33	2.39 (−1.46 to 6.38)	0.23

Models adjusted according to model B (Table 2). Estimates are presented as a percentage change in average relative telomere length for each kg/m^2^ BMI increase or for a SD increase in weight gain during pregnancy. Categorical weight gain defined according to the Institute of Medicine guidelines [48]. Insufficient and excessive gestational weight gain in relation to maternal pre-pregnancy BMI (for underweight: total weight gain < 13 and > 18 kg; normal weight: total weight gain < 11.5 and > 16.0 kg; for overweight: total weight gain < 7.0 and > 11.5 kg; for obesity: total weight gain < 5.0 and > 9.0 kg, respectively)

**Table 5:** Sensitivity analyses

	Cord blood telomere length			Placental telomere length		
	n	Percentage change (95 % CI)	*P* value	n	Percentage change (95 % CI)	*P* value
Model B	743	−0.50 (−0.83 to −0.17)	0.003	702	−0.66 (−1.06 to −0.25)	0.002
Excluding non-European	658	−0.47 (−0.82 to −0.11)	0.01	621	−0.50 (−0.93 to −0.06)	0.03
Excluding BMI < 18.5 kg/m^2^	714	−0.43 (−0.78 to −0.07)	0.02	675	−0.61 (−1.03 to −0.17)	0.006
Excluding cesarean sections	710	−0.50 (−0.84 to −0.16)	0.004	673	−0.59 (−1.00 to −0.18)	0.005
Excluding pregnancy complications	665	−0.53 (−0.89 to −0.17)	0.004	628	−0.74 (−1.19 to −0.30)	0.001
Excluding all above	542	−0.44 (−0.87 to −0.00)	0.05	513	−0.45 (−0.98 to 0.08)	0.09

Estimates are presented as a percentage change in average relative telomere length for each kg/m^2^ BMI increase

All models were adjusted according to Model B (Table 2)

## Discussion

Persons of the same age vary greatly with regards to telomere length and this variation is present from early life. The key finding of our paper is that pre-pregnancy BMI is associated with shorter newborn cord blood and placental telomeres. These associations remained unchanged after adjustments for paternal and maternal age at birth, maternal education, and newborn gender, birth weight, and gestational age. Our findings shed light on the pre-pregnancy effects of maternal BMI on the next generation. Indeed, our data showed that, for each BMI unit increase, average relative cord blood and placental telomeres were 0.50 % and 0.66 % shorter, respectively. The telomere loss in newborns of obese mothers may increase the risk for chronic diseases in adulthood. As we used a real-time PCR method we are not able to provide absolute values of telomere lengths to estimate the effects of our decline based on absolute values as measured, for instance, using terminal restriction fragments. Nevertheless, an estimation can be based on available data from mean umbilical cord blood telomere lengths (measured using TRF), leading to an estimated value of approximately 10 kb [20, 21, 52, 53], indicating a decrease of 0.50 % leads to a loss of approximately 50 bp in cord blood telomere length for each maternal BMI point increase. Based on longitudinal studies, an annual loss between 32.2 and 45.5 bp is estimated in adult leukocytes [54], indicating that each maternal pre-pregnancy BMI point increase is equivalent to a loss of 1.1 to 1.6 telomeric year equivalence in adulthood (based on telomere attrition of 32.2–45.5 bp/year). This illustrates the public health significance of our findings, as newborns from obese mothers compared with newborns from normal weight mothers were biologically approximately 12 to 17 years older, based on telomeric year equivalence in adulthood.

Longitudinal studies have shown that telomere attrition is greatest during early life. Experimental studies in zebra finches show that telomere length in early life is predictive of longevity [55]. Therefore, our results of maternal BMI on newborn telomere length comprise an important public health finding.

Epidemiologic and animal studies indicate associations between pre-pregnancy maternal obesity and cardiovascular diseases and metabolic disorders in the offspring [45, 46]. Maternal obesity during the first trimester of pregnancy has been associated with a relative risk of childhood obesity of 2.3 (95 % CI, 2.0–2.6) at the age of 4 years [43]. A suggested mechanism that could underlie these relationships is *in utero* fetal programming by nutritional stimuli [38]. Fetuses have to adapt to the supply of nutrients crossing the placenta, which may permanently change their physiology and metabolism.

Our findings in newborns support the association between BMI and telomere length in adulthood. Meta-analytical evidence suggests that leukocyte telomere length is inversely associated with BMI in adulthood [33]. In adult women, Valdes et al. [56] reported on the average 240 bp shorter telomeres in obese women BMI > 30 compared with lean women which corresponds to a age difference of 8.8 years. The role of the enzyme telomerase in the association between increased BMI and shortened telomeres is less well understood. Epel et al. [57] described low telomerase activity with increased BMI in 62 adult healthy women, which may be an important factor for the observed shortened telomeres in relation with body weight. Whether altered telomerase activity in mothers due to increased BMI also indicates altered neonatal telomerase activity remains unclear and might be an interesting topic of research. Obesity increases systemic inflammation and generation of reactive oxygen species (ROS) in fat cells [58–60]. These high levels of ROS resulting in higher oxidative stress might explain accelerated shortening of telomeres in addition to cellular replication [61, 62]. As telomeres contain G-rich fragments that are highly sensitive to ROS, these higher levels of oxidative stress can lead to breakage of DNA and a more rapid decline in telomere length [63]. Higher maternal oxidative stress and inflammation status due to increased obesity might generate a higher inflammatory and oxidative stress intrauterine environment for the developing fetus, influencing telomere biology during the *in utero* life. Recent studies indeed showed that maternal obesity leads to increased oxidative stress in both mothers and newborns. Higher states of oxidative stress were observed in maternal plasma as well as in newborn plasma and placental tissue of obese mothers compared with normal weight mothers [64]. Increased levels of malondialdehyde, superoxide anion, and nitric oxide levels were observed in newborns of obese mothers [64, 65]. These higher levels of oxidative stress have been proposed to induce metabolic alterations that may act as mechanisms in fetal programming [66] and this may provide a link between maternal obesity and shortened telomere lengths in newborns.

In the ENVIR*ON*AGE birth cohort, we observed (in unadjusted analysis), besides an association with pre-pregnancy BMI, longer cord blood and placental telomeres in female newborns compared to male newborns, that telomeres tended to be longer with increased paternal age, and that cord blood telomeres were longer in association with higher maternal education, all of which are in accordance to recent studies [28, 29, 67]. A strength of our study is the large sample size of newborns with matching cord blood and placental tissue, to study telomere length associations. We found consistent results of shorter telomere length in both newborn cord blood and placenta in association with pre-pregnancy BMI. Further, our findings are generalizable as our study population is representative for the gestational segment of the population at large (Additional file 1: Table S1). Our associations remained unchanged after adjustments for different covariates and potential cofounders and persisted across subgroups or after excluding non-European newborns, mothers with pre-pregnancy BMI less than 18.5 kg/m^2^, cesarean sections, and pregnancy complications, further suggesting an independent association. We need to address some limitations of this study. We do not have information on paternal BMI and recent epigenetic effects of paternal weight on newborns have been described [68, 69]. We used a real-time PCR method to determine telomere lengths, which has, in general, higher assay variability compared to the traditionally used TRF method [70, 71]. However, we participated in an inter-laboratory comparison and achieved coefficients of variation of less than 7 %. Further, we acknowledge the fact that variability within the placenta exists, and for our study the intra-placental variability for telomeres was 11 %. Recently, Factor-Litvak et al. [67] showed a strong correlation between newborn telomere length measured in cord blood and both age-adjusted paternal and maternal telomere lengths. As overweight mothers may potentially have shorter telomeres, the association between pre-pregnancy BMI and newborn telomere length might be mediated by maternal telomere lengths. This mediation could not be addressed in the ENVIR*ON*AGE birth cohort at this moment as no data on maternal telomere lengths was available. Finally, we need to acknowledge that other potential important factors that occur during pregnancy, such as newborn telomerase activity and alteration of oxidative stress-related markers in mothers and newborns, and which might influence telomere length at birth, were not measured.

## Conclusions

To our knowledge, this is the first study to report a strong association between maternal pre-pregnancy BMI and telomere lengths in newborns in a large birth cohort. Telomere length in early life predicts life span; therefore, our results on variation in offspring early-life telomere length in association with maternal BMI are a major step forward in unravelling the early life determinants of telomere length. Further, the public health impact is considerable, as in affluent societies approximately 30 % of women of reproductive age are overweight. Our results add to the growing body of evidence that high maternal BMI impacts fetal programming. Maintaining a normal BMI during a women’s reproductive age may promote molecular longevity in the offspring as exemplified by newborn telomere length. In this context, maternal overweight and obesity might be one of the most preventable environmental factors that may increase life expectations of newborns and may impact overall quality of life by decreasing comorbidities in adulthood.

## Additional file

Additional file 1: Text S1. Telomere and single copy-gene reaction mixture and PCR cycling conditions. **Table S1.** Comparison characteristics of the ENVIR*ON*AGE data with data of the birth register of Flanders (Northern part of Belgium). **Figure S1.** Flowchart of included mother newborn pairs. **Figure S2.** Pearson correlation between average relative telomere lengths in cord blood and placental tissue. (DOCX 237 kb)

## Abbreviations

BMI: body mass index
CI: confidence interval
CV: coefficient of variation
ENVIR*ON*AGE: Environmental Influence on Ageing in Early Life birth cohort
qPCR: quantitative real-time PCR
ROS: reactive oxygen species
SD: standard deviation
T/S ratio: telomere to single-copy gene ratio
TRF: terminal restriction fragment
