# Supplementary material for: Maternal pre-pregnancy body mass index and newborn telomere length
Source: BMC Med. 2016 Oct 18;14:148. doi: 10.1186/s12916-016-0689-0 (PMC5067896; doi:10.1186/s12916-016-0689-0)
Supplement: Additional file 1: Text S1. — Telomere and single copy-gene reaction mixture and PCR cycling conditions. Table S1. Comparison characteristics of the ENVIRONAGE data with data of the birth register of Flanders (Northern part of Belgium). Figure S1. Flowchart of included mother newborn pairs. Figure S2. Pearson correlation between average relative telomere lengths in cord blood and placental tissue. (DOCX 237 kb) [file 12916_2016_689_MOESM1_ESM.docx]

**Supplemental information**

# Newborn telomere length and maternal pre-pregnancy body-mass index

Dries S. Martens, Michelle Plusquin, Wilfried Gyselaers, Immaculata De Vivo, Tim S. Nawrot

**Table of contents**

Text S1: Telomere and single copy-gene reaction mixture and PCR cycling conditions.

Table S1: Comparison characteristics of the ENVIRONAGE data with data of the birth register of Flanders (Northern part of Belgium).

Figure S1: Flowchart of included mother newborn pairs.

Figure S2: Pearson correlation between average relative telomere lengths in cord blood and placental tissue.

Text S1. Telomere and single copy-gene reaction mixture and PCR cycling conditions.

Telomere reaction mixture contained 1x QuantiTect SYBR Green PCR master mix (Qiagen, Inc., Venlo, the Netherlands), 2 mM dithiothreitol (DTT), 300 nM telg primer (ACACTAAGGTTTGGGTTTGGGTTTGG GTTTGGGTTAGTGT) and 900 nM telc primer (TGTTAGGTATCCCTATCCCTATCCCTATCCCTATCCCT AACA). Used cycling conditions were: 1 cycle at 95°C for 10 min, followed by 2 cycles at 94°C for 15 sec and 49°C for 2 min and 30 cycles at 94°C for 15 sec, 62°C for 20 sec, and 74°C for 1 min and 20 sec. The single-copy gene reaction mixture contained 1x QuantiTect SYBR Green PCR master mix, 300 nM 36B4u primer (CAGCAAGTGGGAAGGTGTAATCC) and 500 nM 36B4d primer (CCCATTCTATCATCAACGGGT ACAA). Used cycling conditions were: 1 cycle at 95°C for 10 min, followed by 40 cycles at 95°C for 15 sec, and 58°C for 1 min and 20 sec.

Table S1. Comparison characteristics of the ENVIRONAGE data with data of the birth register of Flanders (Northern part of Belgium).

|  | **ENVIR*ON*AGE (n=743)** | **Flanders (n=606 877)** |
| --- | --- | --- |
| **Maternal age, years** | 29.0 (23.0-35.0) | 29.5 (23.5-35.8) |
| **Education** |  |  |
| Low  Middle  High | 11.2% 37.1% 51.7% | 13.1%  40.8%  46.2% |
| **Parity** |  |  |
| 1  2  ≥3 | 56.1% 33.5% 10.4% | 46.9%  34.7%  18.4% |
| **Gender** |  |  |
| Boys | 49.4% | 51.4% |
| **Ethnicity** |  |  |
| European | 88.6% | 87.7% |
| **Birth weight, g** | 3390 (2820-4000) | 3360 (2740-3965) |

This register comprise all birth from Flanders (n=606 877) from 1999-2009.[1]
Values presented as percentage or as median (10-90^th^ centile).


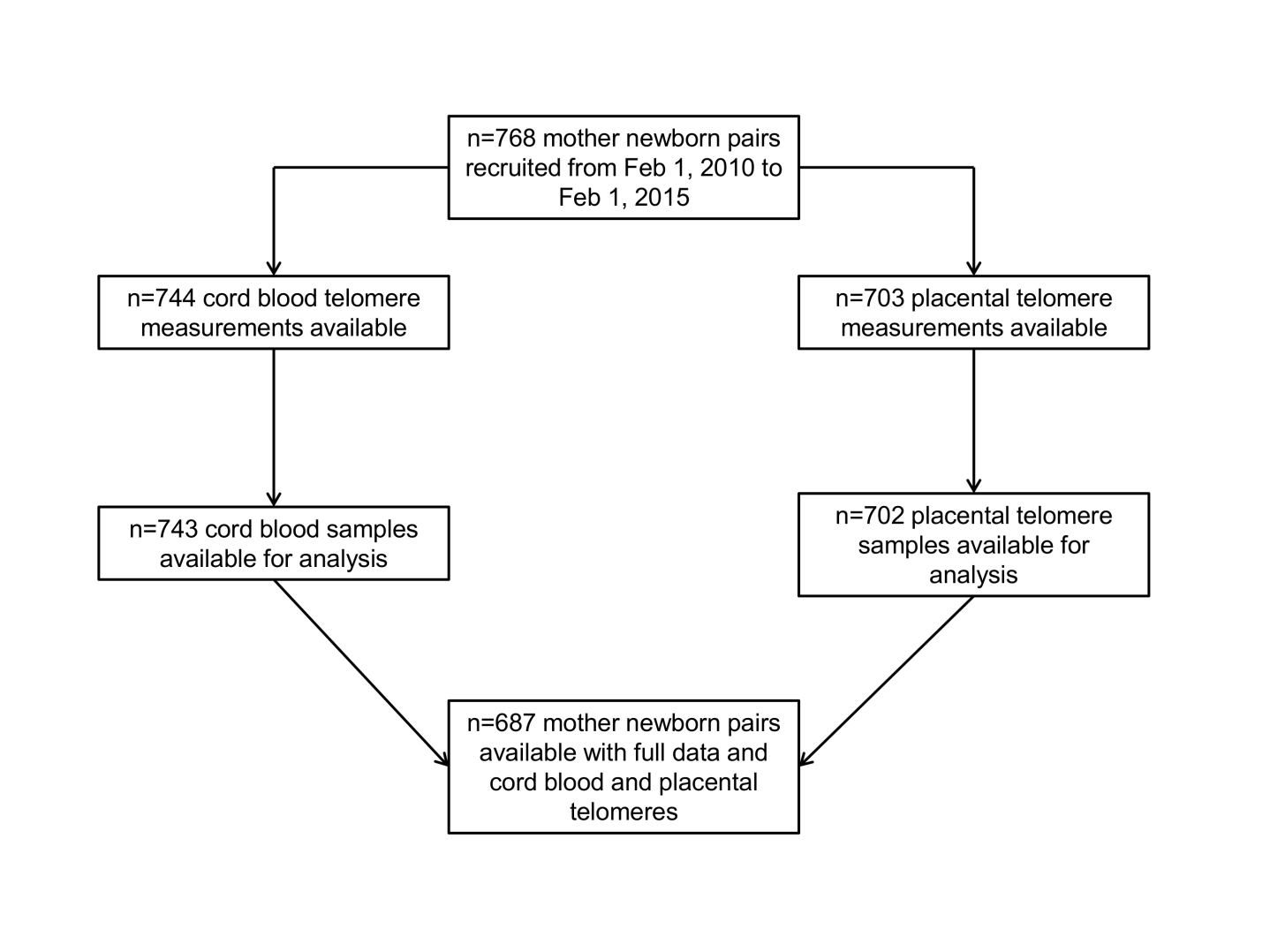


Figure S1. Flowchart of included mother newborn pairs. 768 mother-newborn pairs recruited in the ENVIR*ON*AGE birth cohort from Feb 1, 2010, to Feb 1, 2015. For cord blood analysis, 14 newborns were excluded based on no availability of cord blood or bad quality DNA, additionally 10 newborns were excluded based on non-reproducible telomere measurement and 1 newborn was excluded due to lacking data on maternal weight gain during pregnancy, resulting in 743 mother-newborn pairs to study the association between cord blood telomere length and pre-pregnancy maternal BMI. For placental analysis, 57 newborns were excluded based on no availability of placental tissue or because of bad quality DNA, additionally 8 newborns were excluded based on non-reproducible telomere measurements and 1 newborn was excluded due to lacking data on maternal weight gain during pregnancy, resulting in 702 mother-newborn pairs to study the association with placental telomeres. In total 687 mother-newborn pairs were available with both cord blood and placental telomere measurements. A valid telomere length was defined when variation between replicates for telomere and single-copy gene run where less than 0.3 Ct.

Figure S2. Pearson correlation between average relative telomere lengths in cord blood and placental tissue.

**References**

1. Cox B, Martens E, Nemery B, Vangronsveld J, Nawrot TS. Impact of a stepwise introduction of smoke-free legislation on the rate of preterm births: analysis of routinely collected birth data. BMJ 2013; 346:f441.
